# *PsGSTF3*, an Anthocyanin-Related Glutathione S-Transferase Gene, Is Essential for Petal Coloration in Tree Peony

**DOI:** 10.3390/ijms23031423

**Published:** 2022-01-26

**Authors:** Lulu Han, Lin Zhou, Hongzhu Zou, Meng Yuan, Yan Wang

**Affiliations:** Key Laboratory of Tree Breeding and Cultivation of National Forestry and Grassland Administration, Research Institute of Forestry, Chinese Academy of Forestry, Beijing 100091, China; hanlulu0727@163.com (L.H.); zhoulin1214@163.com (L.Z.); zouhongzhu1123@163.com (H.Z.); yuanmeng00829@163.com (M.Y.)

**Keywords:** *Paeonia suffruticosa*, anthocyanin, transport, glutathione S-transferase

## Abstract

Anthocyanins, as the most important chromogenic substances in flavonoids, are responsible for the red, purple, and blue coloration of flowers. Anthocyanins are synthesized in the cytoplasmic surface of the endoplasmic reticulum (ER) but accumulate predominantly in the vacuole, while glutathione S-transferases (GSTs) are considered to be mainly responsible for the transport process. Our previous studies showed that the expression of *PsGSTF3* was positively correlated with anthocyanin content in tree peony tissues, which is a key candidate gene for anthocyanin accumulation. Here, we successfully cloned and characterized full-length *PsGSTF3* containing three exons and two introns. Subcellular localization showed that *PsGSTF3* was localized in the nucleus and ER membrane. Functional complementation of the *Arabidopsis* transparent testa19 (*tt19*) mutant indicated that *PsGSTF3* was responsible for the transport of anthocyanins but not of proanthocyanidins (PAs). Virus-induced gene silencing (VIGS) of *PsGSTF3* not only led to a decrease in anthocyanin accumulation but also caused a reduction of structural genes in the anthocyanin biosynthesis pathway (ABP) to varying degrees. Heterologous overexpression of *PsGSTF3* was found to increase the anthocyanin accumulation in tobacco petals. Furthermore, the yeast two-hybrid (Y2H) assay showed that *PsGSTF3* interacted with PsDFR, which together contributed to the coloration of petals. In conclusion, these results demonstrate that *PsGSTF3* encodes an important GST transporter of anthocyanin in tree peony petals and provides a new perspective for the associated transport and regulatory mechanisms.

## 1. Introduction

Anthocyanins, as the most important chromogenic substances in flavonoids, are a group of water-soluble pigments responsible for the red, purple, and blue coloration of flowers, fruits, and leaves [1]. Anthocyanins provide visual signals that affect pigmentation, and attract pollinators and seed dispersers [2,3]. In addition, anthocyanins provide defense and protect plants against pathogens and ultraviolet radiation damage [2,4,5]. Interestingly, anthocyanins also play an irreplaceable role in human health due to their strong antioxidant activities [6,7].

The anthocyanin biosynthesis pathway (ABP) is one of the most thoroughly studied secondary metabolic pathways in plants, and is highly conserved [8]. The enzymes that catalyze anthocyanin biosynthesis include *CHS* (chalcone synthase), *CHI* (chalcone isomerase), *F3H* (flavanone 3-hydroxylase), *F3*′*H* (flavonoid 3′-hydroxylase), *F3*′*5*′*H* (flavonoid 3′,5′-hydroxylase), *DFR* (dihydroflavonol 4-reductase), *ANS* (anthocyanin synthase), and *UFGT* (UDP flavonoid glucosyltransferase). Among them, *CHS*, *CHI*, *F3H*, *F3*′*H*, and *F3*′*5*′*H* together comprise the early ABP [8,9], while *DFR*, *ANS*, and *UFGT* are all considered components of the late biosynthetic pathway [2,8,10,11]. Anthocyanin biosynthesis is regulated by the MYB-bHLH-WD40 (MBW) protein complex [10], which is composed of MYB and bHLH transcription factors (TFs) and a WD40 protein. Four R2R3-MYB transcription factors, *PsMYB114L*, *PsMYB12L*, *PsMYB57* and *PsMYB58*, have been verified to upregulate some anthocyanin structural genes and promote the accumulation of anthocyanin in tree peony [11,12,13]. Another MYB, *PqMYB4*, is involved in the negative regulation of anthocyanin biosynthesis in tree peony leaves [14]. In addition, PsMYB12 interacts with bHLH and WD40 proteins in a regulatory complex that directly activates *PsCHS* expression, which is specific to petal blotches [15]. In summary, structural genes and TFs together constitute a molecular regulatory network for ABP in plants [9].

However, anthocyanin is biosynthesized on the cytosolic surface of the endoplasmic reticulum (ER) and then transported to the acidic vacuole for storage before ultimately presenting brilliant colors [2,16]. In recent years, studies have begun to clarify some problems in the transport of anthocyanin in plants. Two types of anthocyanin transport mechanisms in plants have been proposed: (1) vesicle-mediated transport based on the vesicle-like structures accumulating anthocyanin with the central vacuole [17] and (2) translocator-mediated transport, including glutathione S-transferase (GST), adenosine triphosphate (ATP)-binding cassette (ABC) proteins, and multidrug and toxic extrusion (MATE) transporters [18,19,20]. Here, we focus on the GST family, which represents a ubiquitous and complex superfamily of multifunctional dimeric enzymes [19] and catalyzes the combination GSH with toxic heterologous substances or oxidation products to promote metabolism and regional isolation or elimination [21]. In plants, some GSTs ubiquitously participate in the accumulation of secondary metabolites, signal transduction, and responses to biotic and abiotic stresses [21,22,23]. Through multiple analyses and verifications of functions, GSTs were found to be involved in the transport and accumulation of anthocyanin. This role was first demonstrated in maize by its mutant *Bronze*-*2* (*Bz2*, a GST-encoding gene), which accumulates anthocyanin only in the cytoplasm [24]. Additionally, anthocyanin-related GSTs have been found in *Arabidopsis* transparent testa19 (*tt19*) [25] and petunia *AN9* [26]; these are mutants lacking GST related to anthocyanin transport, which often leads to an anthocyanin-less phenotype with a reduced amount of pigment. Such GSTs have also been found in fruit crops, such as *MdGSTF6* in apple [27], *LcGST4* in litchi [28], *AcGST1* in kiwifruit [29], *PpGST1* in peach [30], and *FvRAP* in strawberry [31]. GSTs were confirmed to be involved in anthocyanin sequestration in these fruits. Recently, anthocyanin-related GSTs have also been reported in ornamental flowers. For example, in cineraria, the expression of *ScGST3* was found to be directly proportional to the anthocyanin content in the tissue [32]; in cyclamen, *CkmGST3* complemented the anthocyanin-less phenotype of the *Arabidopsis tt19* mutant and restored the accumulation of anthocyanins [33]; in carnation, introducing the *DcGSTF2* gene into the epidermal cells of carnations with pale pink petals caused the transformed cells to become deep pink [34]. A comprehensive summary is shown in Table S1.

Peony (*Paeonia suffruticosa*) is among the most valued traditional ornamental flowers in China and has a variety of colors. For a long time, flower color has been an important ornamental characteristic for plants, and for horticulturists, color is also an important breeding goal. The composition of flavonoids in petals is the basis for the formation of flower color in peony, and purple–red flowers mainly represent anthocyanin [35]. Previous studies have investigated the biosynthesis and transcriptional regulation of anthocyanin in tree peony [11,12,13,15,36,37,38]; however, little is known about the mechanism underlying the vacuolar sequestration of anthocyanin in peony petals.

In our previous study, 54 GSTs were systematically identified, through a combination of bioinformatics approaches, from tree peony petal transcriptome databases [39]. A Phi (F) class GST member, *PsGSTF3*, was speculated to be a candidate participant in anthocyanin transport and the promotion of pigment accumulation, exhibiting a strong positive correlation with anthocyanin content in different tissues [39]. Here, we used different approaches to demonstrate the functionality of *PsGSTF3* as an anthocyanin transporter. Subcellular localization demonstrated that *PsGSTF3* was localized on the nucleus and ER membrane. Overexpression of *PsGSTF3* in the *Arabidopsis tt19* mutant compensated for defective anthocyanin pigmentation. Moreover, *PsGSTF3* that was ectopically transformed into tobacco was able to deepen the colors of flowers, and silencing of *PsGSTF3* affected anthocyanin accumulation in petals of tree peony. In addition, the yeast two-hybrid (Y2H) experiment showed that *PsGSTF3* interacted with PsDFR. These results indicated that *PsGSTF3* encodes an important anthocyanin transporter that affects anthocyanin accumulation in tree peony. These findings will facilitate our in-depth understanding of the formation mechanism of tree peony flower colors.

## 2. Results

### 2.1. Cloning and Characterization Analysis of PsGSTF3

**Figure 1:**
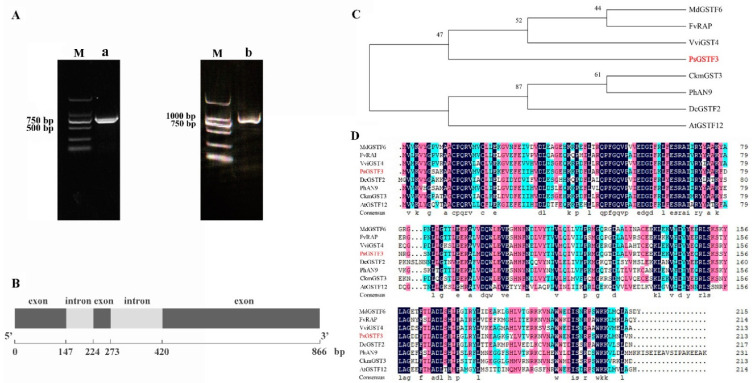
Sequence and phylogenetic analysis of *PsGSTF3*. (**A**) Polymerase chain reaction (PCR) amplification products of *PsGSTF3*: M—DNA marker DL2000; a—cDNA fragment; b—genomic DNA fragment. (**B**) Sequence structure of *PsGSTF3*. (**C**) Phylogenetic tree analysis of glutathione S−transferases (GSTs). The protein sequences are listed in Table S3. (**D**) Multiple alignment of the deduced *PsGSTF3* amino acid sequences with its homologs. The different colors represent different identity.

In our previous studies, *PsGSTF3* was inferred to be involved in anthocyanin transport and accumulation. The genomic DNA and cDNA of *P. suffruticosa* ‘Zhao fen’ were used as templates for amplification, and sequences with lengths of 866 and 642 bp were obtained, respectively (Figure 1A; Table S2). By comparing the genomic DNA and cDNA sequences, we found that *PsGSTF3* contains three exons and two introns (Figure 1B).

*PsGSTF3* belongs to the Phi subclass, which also contains some anthocyanin-related GSTs in dicotyledons such as *MdGSTF6* (*Malus domestica*), *FvRAP* (*Fragaria vesca*), *VviGST4* (*Vitis vinifera*), *CkmGST3* (*Cyclamen persicum*), *PhAN9* (*Petunia hybrida*), *DcGSTF2* (*Dianthus caryophyllus*) and *AtGSTF12* (*Arabidopsis thaliana*) [40]. In the phylogenetic tree, *PsGSTF3* is clustered in the same branch with MdGSTF6, FvRAP, and VviGST4 firstly, and the multiple sequence alignment analysis showed that *PsGSTF3* has higher similarity with them (Figure 1C,D). In summary, *PsGSTF3* is a strong candidate for an anthocyanin transporter involved in the coloration of tree peony petals.

### 2.2. Subcellular Localization of PsGSTF3

**Figure 2:**
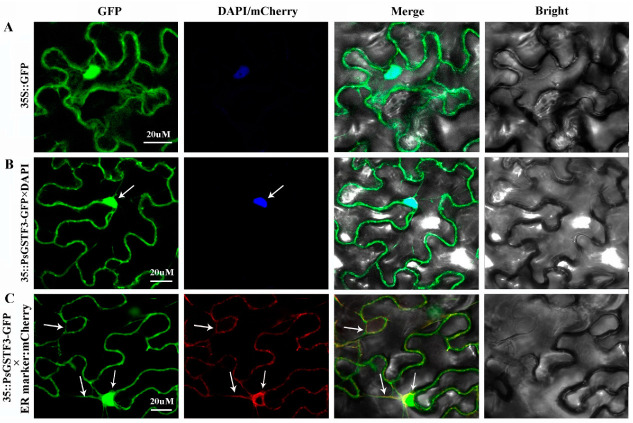
Subcellular localization of *PsGSTF3* in *Nicotiana benthamiana*. (**A**) Subcellular localization in *N. benthamiana* infected with 35S::GFP and DAPI. (**B**) *N. benthamiana* infected with 35S::PsGSTF3−GFP and DAPI. (**C**) *N. benthamiana* infected with 35S::PsGSTF3−GFP and ER−located marker labeled with mCherry.

Subcellular localization of *PsGSTF3* was carried out using fluorescent reporter genes (GFP). A 35S::PsGSTF3-GFP recombinant vector was constructed and introduced into tobacco leaf epidermal cells using 35S::GFP as the negative control, and GFP fluorescence was observed with a laser confocal microscope (ZEISS LSM880 Airyscan FAST+NLO, Germany). Based on the observed co-localization with the marker proteins (nucleus marker-DAPI and ER marker-mCherry), 35S::PsGSTF3-GFP fluorescence was present in both nucleus and ER, whereas 35S::GFP fluorescence showed a diffuse distribution throughout the whole cell (Figure 2). Thus, we speculated that the *PsGSTF3* protein is located on the nucleus and ER, and may play a role in those areas.

### 2.3. Heterologous Expression of PsGSTF3 in the tt19 Mutant

**Figure 3:**
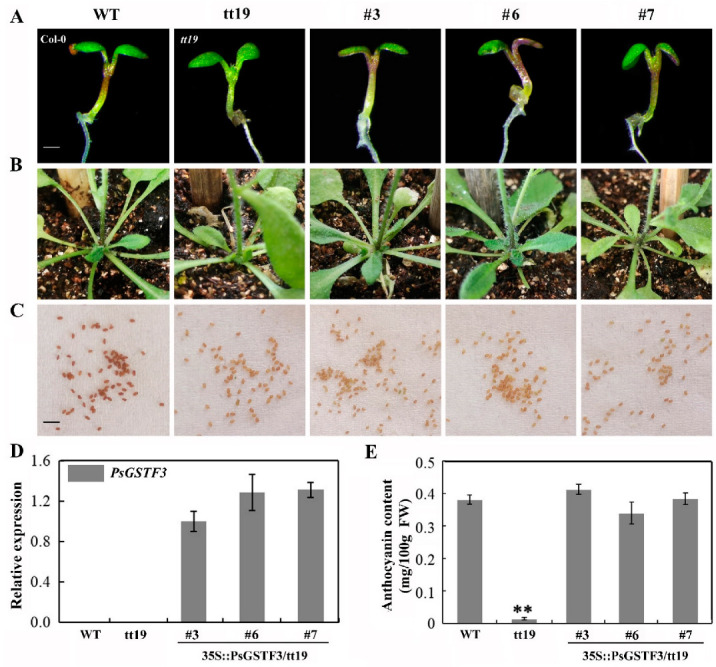
Functional complementation of *Arabidopsis* transparent testa19 (*tt19*) mutant with *PsGSTF3*. (**A**) Phenotypic characterization of the 7−day−old *Arabidopsis* wild type (WT), *tt19,* and transgenic lines (35S::*PsGSTF3*/*tt19*, Lines 3, 6 and 7) grown on the Murashige and Skoog (MS) medium with 6% sucrose using a stereomicroscope (LEICA M205 FA, Germany). (**B**) Phenotypic characterization of the corresponding adult plants. (**C**) Phenotypic characterization of *Arabidopsis* seed coats. White bar: 1 mm; black bar: 2 mm. (**D**) Expression analysis of *PsGSTF3* in 35S::*PsGSTF3*/*tt19* transgenic lines (Lines 3, 6, and 7), as well as the WT and *tt19* mutant. (**E**) Anthocyanin content in the WT, *tt19*, transgenic lines 3, 6, and 7. Data are presented as the means (±SD) from three independent replicates. The asterisks denote significant differences according to a one−way analysis of variance (ANOVA) (** *p* < 0.01).

*PsGSTF3* is a homolog of AtGSTF12 that encodes an anthocyanin transporter [41]. To explore the function of *PsGSTF3* in vivo, a molecular complementation experiment was performed using *Arabidopsis tt19*, which is a knockout mutant of the anthocyanin transporter GST [41]. Three independent transgenic plants (Lines 3, 6 and 7) with higher expression levels of *PsGSTF3* were screened via a reverse transcription–polymerase chain reaction (RT–PCR) experiment from 16 positive transgenic lines (Figure S1), which were used for subsequent analyses (Figure 3D). In the 7-day-old seedlings and adult plants, the hypocotyl and basal regions of stems in the *tt19* mutant remained green, while those of the three transgenic lines recovered purple pigmentation just like wild type (WT) (Figure 3A,B). Furthermore, the quantification of anthocyanin contents was consistent with the visual inspection, with numbers about 29, 24, and 27 times higher in Line 3, Line 6, and Line 7, respectively, than those in *tt19* (Figure 3E). However, the 35S::PsGSTF3-PHB transgenic lines did not rescue the pale brown seed coats in *tt19* (Figure 3C). This result indicated that *PsGSTF3* does not play a role in the seed coat pigmentation of the *tt19* mutant and does not participate in PA accumulation. In conclusion, based on the *PsGSTF3* functional complementarity in *Arabidopsis tt19*, we determined that *PsGSTF3* is involved in anthocyanin, but not PA, transport in tree peony.

### 2.4. Virus-Induced Gene Silencing (VIGS) of PsGSTF3 Influences Tree Peony Petal Coloration

**Figure 4:**
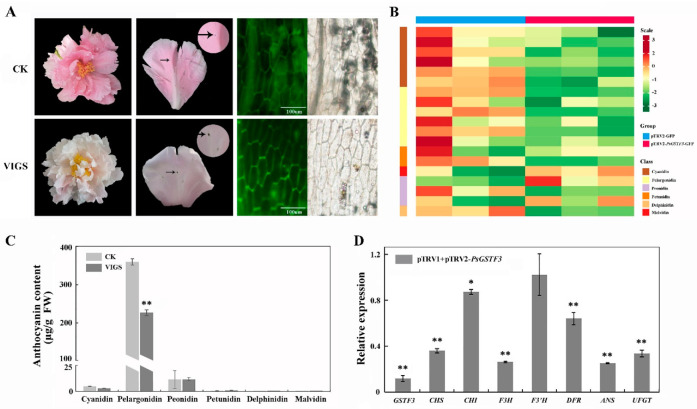
Functional analysis of the *PsGSTF3* gene by TRV (tobacco rattle virus) −based virus−induced gene silencing (VIGS). (**A**) Comparisons between pTRV1/pTRV2−GFP and pTRV1/pTRV2−*PsGSTF3*−GFP treated groups with respect to flowers at the full opening stage; fluorescence signals of petals were imaged by fluorescence microscope. The photograph was taken at 7 d after infiltration. (**B**) Heatmap analysis of each anthocyanin component. The contents were log-transformed and used to generate a heatmap with the TBtools software package. (**C**) Anthocyanin contents of petals via two experimental treatments. CK—petals injected with pTRV1/pTRV2−GFP; VIGS—petals injected with pTRV1/pTRV2−*PsGSTF3*−GFP. (**D**) Relative expression of *PsGSTF3* and structural genes in ABP in the petals at 7 d after infiltration. The expression in the empty−vector (pTRV1/pTRV2−GFP) infiltrated petals was used as the calibrator (set as 1). Data are presented as the means (±SD) from three biological replicates. The asterisks denote significant differences according to a one−way analysis of variance (ANOVA) (* *p* < 0.05; ** *p* < 0.01).

The VIGS system was used to demonstrate whether *PsGSTF3* is essential for tree peony petal coloration. Infection solutions mixed in equal volumes with pTRV1/pTRV2-GFP and pTRV1/pTRV2-PsGSTF3-GFP were transiently infiltrated into the unpigmented or slightly pigmented tight buds of *P. suffruticosa* ‘Zhao fen’. At 7 d after infection, less pigmentation overall was observed in the tree peony petals injected with pTRV1/pTRV2-PsGSTF3-GFP, especially near the injection site, with no significant changes found in the petals injected with pTRV1/pTRV2-GFP (Figure 4A). Meanwhile, GFP detection was performed on the petals infected with pTRV1/pTRV2-GFP and pTRV1/pTRV2-PsGSTF3-GFP solutions via a fluorescence microscope (Olympus BX51, Tokyo, Japan). We also found that the petals treated with VIGS showed green fluorescence signals (Figure 4A).

Further, ultra-performance liquid chromatography (UPLC) was used to determine the anthocyanin contents in the two experimental groups. The chromatograms showed that the components of the anthocyanin were well separated, and six common anthocyanin classes (Cyanidin, Pelargonidin, Peonidin, Petunidin, Delphinidin and Malvidin) were measured. In *P. suffruticosa* ‘Zhao fen’ petals, the Pelargonidin class had the highest content, followed by the Peonidin class, while the contents of the Petunidin, Delphinidin, and Malvidin classes were extremely low (Figure 4B,C). In addition, the anthocyanin content of the petals injected with pTRV1/pTRV2-PsGSTF3-GFP was significantly lower compared to the corresponding injection in pTRV1/pTRV2-GFP petals (Figure 4B; Table S4), which correlated well with the reduced expression level of *PsGSTF3* (Figure 4D). Interestingly, knockdown of *PsGSTF3* significantly downregulated the expression of *PsGSTF3* and also affected the expression of structural genes (except *F3*′*H*) in the ABP to varying degrees (Figure 4D). In short, these results showed that the knockdown of *PsGSTF3* can affect anthocyanin accumulation directly and that *PsGSTF3* plays an irreplaceable role in anthocyanin transport and accumulation in tree peony petals.

### 2.5. Overexpression of PsGSTF3 Increased Anthocyanin Accumulation in Tobacco

**Figure 5:**
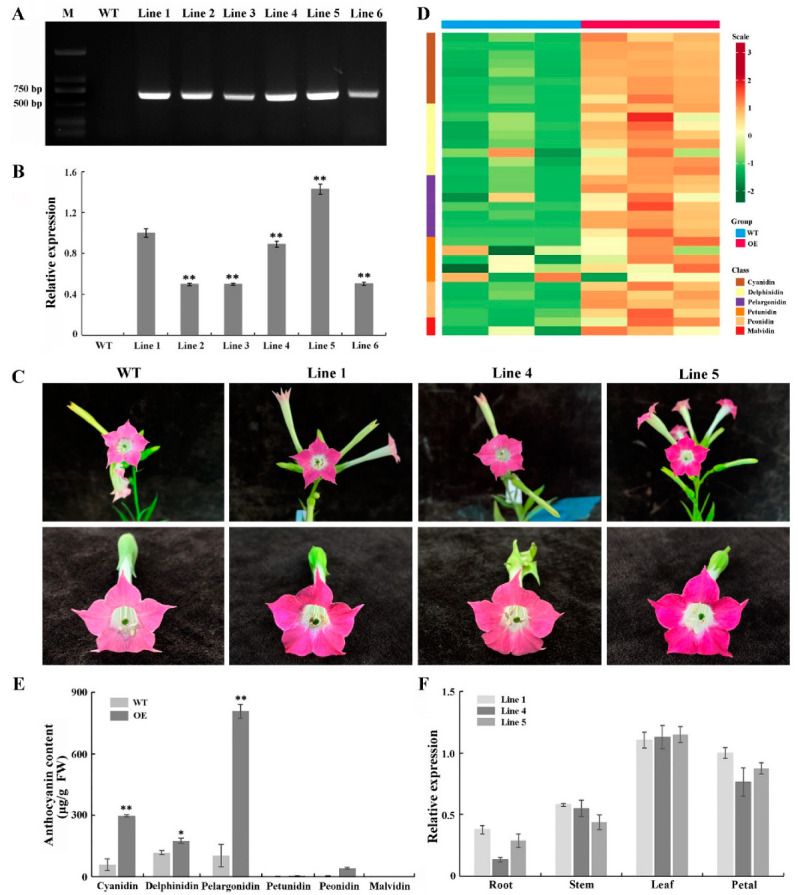
Functional analysis of tobacco lines overexpressing the *PsGSTF3* gene. (**A**) Electropherogram of positive PCR detection in transgenic tobacco lines. (**B**) The results of quantitative real-time polymerase chain reaction (qRT−PCR) detection in transgenic tobacco lines. (**C**) Phenotypic characterization of tobacco flowers between the control group and transgenic lines. (**D**) Heatmap analysis of each anthocyanin component. The contents were log-transformed and used to generate a heatmap with the TBtools software package. (**E**) Anthocyanin contents of tobacco petals via two experimental treatments: WT—petals of the wild type; OE—petals of the transgenic lines. (**F**) Temporal and spatial expression patterns of *PsGSTF3* in the three transgenic tobacco lines. Data are the means (±SD) from three biological replicates. The asterisks denote significant differences according to a one−way analysis of variance (ANOVA) (* *p* < 0.05; ** *p* < 0.01).

To further characterize the function of *PsGSTF3*, a 35S::PsGSTF3-PHB vector was ectopically introduced into the tobacco. Six independent transgenic tobacco lines that overexpressed *PsGSTF3* were screened in a hygromycin-resistant medium and were then cultured under the same conditions. RT–PCR analysis showed that the transformed *PsGSTF3* gene was detected in all transgenic lines but was nonexistent in the control group (WT) (Figure 5A). Furthermore, the quantitative real-time polymerase chain reaction (qRT–PCR) analysis revealed that the expression level of *PsGSTF3* in transgenic plants was significantly higher than that in the control group, especially in Line 1, 4 and 5 (Figure 5B). Therefore, these three lines were used for further experiments.

Notably, compared to the control group, the petal pigmentation of the transgenic tobacco harboring *PsGSTF3* was markedly deeper (Figure 5C). To confirm whether the deeper flower color could be attributed to the increased levels of pigment synthesized from the anthocyanin pathway, the anthocyanins were determined qualitatively and quantitatively via UPLC. The results showed that most components of anthocyanins were upregulated to varying degrees in transgenic lines compared to the control group (Figure 5D), especially the Cyanidin, Delphinidin, and Pelargonidin classes, which showed significant differences (Figure 5E). Most notably, the extremely significant increase in the content of cyanidin-3-*O*-rutinoside and pelargonine-3-*O*-rutinoside produced this difference (Table S5). In addition, the expression patterns of *PsGSTF3* in the three transgenic tobacco lines were analyzed by qRT-PCR. *PsGSTF3* was detected in different tissues, with the highest expression level in the leaves, followed by petals (Figure 5F). In short, the expression of *PsGSTF3* in the transgenic tobacco was closely related to increased pigmentation in the petals.

### 2.6. Predicted Protein Interaction Networks

**Figure 6:**
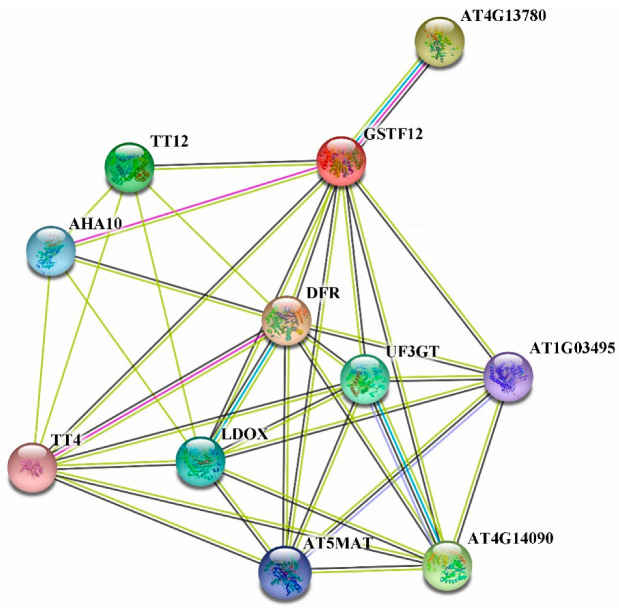
Predicted AtGSTF12 protein interaction networks. Lines of different colors indicate distinct evidence used to predict the network of interactions.

It is known that *PsGSTF3* and AtGSTF12 are homologous, so we used the STRING11.0 online software (https://cn.string-db.org/, accessed on 23 December 2021) to determine which proteins may interact with *PsGSTF3* by calculating the interaction network with AtGSTF12 as the target gene (Figure 6). Analysis of the AtGSTF12 protein interaction networks revealed 10 proteins that interacted, or had a co-expression relationship, with AtGSTF12; the functions and detailed information are shown in Table 1. In addition, we found that eight of these genes played important roles in ABP. Subsequently, these *Arabidopsis* sequences were employed as queries in a Basic Local Alignment Search Tool (BLAST) search of the transcriptome database (Accession number: SRP235658) using the BioEdit software (Version 7.0.9; Micro Focus, Newbury, UK). After the final alignment, seven candidate sequences from tree peony were obtained, including PsMATE, PsCHS, PsDFR, PsANS, PsUF3GT, PsUGT and Ps3AT.

### 2.7. Interaction Detection between PsGSTF3 and Proteins That Related to Anthocyanins

**Figure 7:**
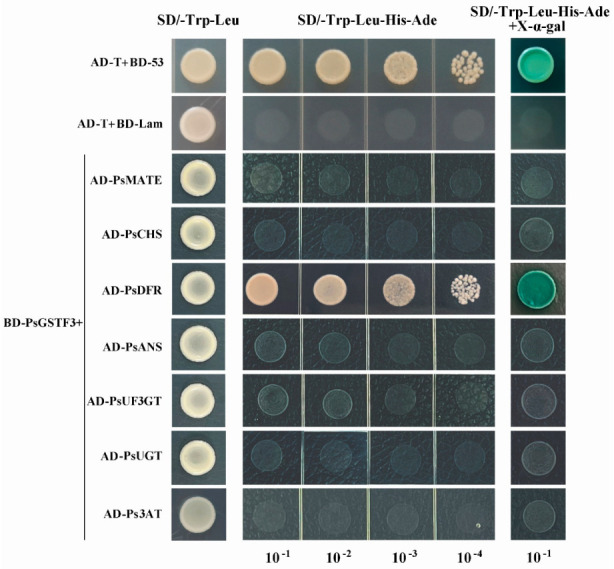
Interaction detection between *PsGSTF3* and seven anthocyanin-related proteins. AD and BD represent empty pGADT7 and pGBKT7 vectors, respectively. SD/−Trp−Leu represents the synthetic dextrose (SD) medium lacking tryptophan and leucine. SD/−Trp−Leu−His−Ade represents the SD medium lacking tryptophan, leucine, histidine, and adenine. The GAL4 activation domain expressed by AD−T and BD−P53 was used as a positive control. AD−T and BD−Lam were used as the negative control.

To determine whether *PsGSTF3* would interact with the seven screened proteins related to anthocyanins, a Y2H assay was performed. The full-length *PsGSTF3* sequence was used as the bait. Firstly, we tested whether there was an autoactivation of *PsGSTF3*. As shown in Figure S2, pGBKT7 (BD)-PsGSTF3 and the pGADT7 (AD) empty vector grew normally on the synthetic dextrose (SD)/-Trp-Leu solid medium (without tryptophan and leucine) but could not grow on the SD/-Trp-Leu-His-Ade solid medium (without tryptophan, leucine, histidine, and adenine), indicating no autoactivation of *PsGSTF3*. Subsequently, we performed interaction detection using different combinations of BD-PsGSTF3 and the seven anthocyanin-related proteins that were screened (AD-PsMATE/PsCHS/PsDFR/PsANS/PsUF3GT/PsUGT/Ps3AT). At the same time, AD-T/BD-P53 and AD-T/BD-Lam were used as the positive and negative control, respectively. The results showed that all different combinations grew well on the SD/-Trp-Leu solid medium, indicating that the two plasmids were co-transformed successfully. However, only AD-T/BD-P53 and AD-PsDFR/BD-PsGSTF3 grew normally on the SD/-Trp-Leu-His-Ade selective solid medium, while the other co-transformed combinations did not grow on the same medium, suggesting that *PsGSTF3* interacted with PsDFR (Figure 7).

## 3. Discussion

Flower color is one of the most important aesthetic traits of ornamental plants, as well as the most important breeding goal of horticulturists. Anthocyanins not only contribute to the red–purple coloration of horticultural plant organs but also are vital to human health and the bioactive substances in plant life. Therefore, research on the regulation of anthocyanin synthesis and transport has far-reaching significance.

Although the structural genes and regulatory genes of anthocyanin biosynthesis in tree peonies have been extensively studied, there are few works on anthocyanin transport after synthesis. To date, the mechanisms underlying the intracellular transport of anthocyanins have been partially elucidated. The delivery of anthocyanins from synthesis to accumulation requires a vesicle trafficking-mediated model [19,42,43] or a transporter-mediated (e.g., MATE, ABC, and GST) model [18,19,20]. GSTs represent a ubiquitous and complex superfamily of multifunctional dimeric enzymes involved in the key metabolic steps of many eukaryotes [19].

The involvement of GSTs in the trafficking and accumulation of anthocyanins was confirmed in maize, *Arabidopsis,* and petunia, as the loss-of-function mutants in these plants cannot accumulate anthocyanins [26,44,45]. Moreover, the transcription abundance of GSTs is reportedly correlated with fruit pigmentation in some horticultural crops, such as apple [27], lychee [28], peach [30], grape [46], and strawberry [31]. In recent years, related studies have also been published on ornamental plants, including camellia [47], cineraria [32,42], carnation [34], cyclamen [33], lily [43], and poinsettia [44] (Table S1). Fortunately, in our previous research, we screened a GST (*PsGSTF3*) gene regarded as a candidate participant in anthocyanin transport and the promotion of pigment accumulation. In this study, we successfully cloned the *PsGSTF3* gene from the petals of *P. suffruticosa* ‘Zhao fen’ that had full-length genomic DNA and cDNA of 866 bp and 642 bp, respectively, and contained three exons and two introns (Figure 1A,B). According to relevant statistics, among the various GST subclasses, intron–exon organization is well categorized and shows similar gene structures, such that most members belonging to the Phi subclass are composed of three exons and two introns [45,48,49,50,51]. Unsurprisingly, *PsGSTF3* was completely suitable for the structural characteristics of the Phi subclass, which may be related to the potential functions of *PsGSTF3* gene.

Subcellular localization analysis can provide important clues for understanding protein functions. In *Arabidopsis*, TT19-GFP is localized not only in the cytoplasm and nuclei, but also on the tonoplast [41]. In contrast, maize Bz2 was confirmed to loosely bind to membranes [52], but it remains unclear with which organelle membrane Bz2 is associated. In this study, *PsGSTF3* was localized on the nucleus and ER membrane (Figure 2). It is well known that anthocyanins are synthesized on the cytosolic surface of the ER and then transported into the vacuole for storage. Sun (2012) [41] reported that cyanidin might be the primary target to which TT19 binds around the cytoplasmic surface of the ER in *Arabidopsis*. The TT19-bound flavonoids were further modified by glycosylation and acylation. Then, the acylated anthocyanins were released from TT19 and taken up by tonoplast-localized transporters and ultimately sequestered into the vacuole [53,54]. The flavonoid transport mechanisms in plants are diverse and redundant to adapt to changing environmental conditions [55]. Thus, we hypothesized that *PsGSTF3* may be a component of the metabolon on the ER and bind to anthocyanins covalently to form a conjugate for labelling anthocyanins, thereby enabling anthocyanins to be identified and transported accurately.

The function of GST in anthocyanin transport was successively verified via the application of various mutants and functional complementation. It is well known that in the maize *Bz2* (encoding a GST) deletion mutant, anthocyanins can only be detected in the cytosol without being transported into the vacuole [24]. The phenotype of a carnation anthocyanin mutant (*fl3*, encoding an anthocyanin-related GST) was restored after the heterologous expression of maize *Bz2* and petunia *PhAN9* [56]. Interestingly, this study also revealed that a GST member can simultaneously participate in the vacuole sequestration of different flavonoid substances in certain plants. Overexpression of *AtGSTF12* (*Arabidopsis thaliana*), *VviGST4* (*Vitis vinifera*), *AcGST1* (*Actinidia chinensis*), and *RsGST1* (*Raphanus sativus*) in the *Arabidopsis tt19* mutant can restore both the anthocyanin-deficient phenotype in plants and the PA-deficient phenotype in seed coats [27,31,48,57] (Figure 3). However, in this study, *PsGSTF3* had the ability to functionally complement the anthocyanin-deficient phenotype of the *Arabidopsis tt19* mutant but not the PA-deficient phenotype in the seed coat, which was consistent with the results for *MdGSTF6* (*Malus domestica*), *PpGST1* (*Prunus persica*), *GhGSTF12* (*Gossypium hirsutum*), *An9* (*Petunia hybrida*), *LcGST4* (*Litchi chinensis*), *RAP* (*Fragaria ananassa*), etc. [28,29,30,32,33]. In summary, these results indicate that *PsGSTF3* plays an extremely important role in the transport of anthocyanins in tree peony petals.

To clarify the role of *PsGSTF3* in the anthocyanin transport of tree peony, the VIGS technique was used to specifically silence the *PsGSTF3* gene in the petals of *P. suffruticosa* ‘Zhao fen’ and to detect and record the phenotypes of the petals in the blooming period. The VIGS system, which involves TRV1 and TRV2, is an effective tool for use in the functional characterization of genes in vivo [58]. To date, there are few reports on the application of the VIGS system in the field of ornamental plant flower color, including *Rosa rugosa* [59] and *Gerbera jamesonii* [60]. In this study, we used perennial tree peony grown naturally in the field as the experimental material for the first time to explore the key genes that may be involved in anthocyanin transport and obtained preliminary results for gene-silencing efficiency. Compared to the control group, the established optimal VIGS system group resulted in a significant decrease of anthocyanin content in the petals of *P. suffruticosa* ‘Zhao fen’, which was consistent with the significant down-regulation of endogenous *PsGSTF3* transcription abundance (Figure 4). Interestingly, most genes encoding anthocyanin biosynthesis enzymes also showed lower expression in pTRV1/pTRV2-PsGSTF3-GFP treatment (Figure 4C), which was similar to the results for VIGS infection experiment in peach fruits [30]. However, these results were different from those in apple fruits and cineraria leaves, which only caused a significant decrease in the transcriptional levels of silenced gene, whereas the relative expression of other structural genes remained basically unchanged [27,42]. In addition, to further confirm the function of the *PsGSTF3* gene in anthocyanin transport, *PsGSTF3* was first transformed into tobacco, which enhanced the flower coloration of the transgenic tobacco plant (Figure 5). Coincidentally, in the results of anthocyanin content in tree peony and tobacco petals, we found that different kinds of anthocyanins changed to some extent between the treatment and control groups (Figure 4B; Figure 5), indicating that *PsGSTF3* did not specifically bind to a certain anthocyanin. In *Arabidopsis*, the in vitro assays showed that the purified recombinant *TT19* increased the water solubility of cyanidin (Cya) and cyanidin-3-*O*-glycoside (C3G) [41]; in cineraria, *ScGST3* increased the water solubility of C3G and delphinidin-3-*O*-glucosid (D3G) [42]; and in kiwi, *AcGST1* increased the water solubility of cyanidin-3-*O*-galactoside (C3Gal) and cyanidin-3-*O*-xylo-galactoside (C3XG) [29]. In short, these previous research results also indirectly supported our hypotheses.

Undoubtedly, anthocyanin accumulation is coordinately regulated by both biosynthesis and transport. To the best of our knowledge, no publications have yet explored whether there are interactions between structural genes in ABP and anthocyanin transporters. Y2H is an experimental technique that analyzes the interactions of two proteins from the expression product based on the genetic analysis of yeast. In this study, we used the homology of *PsGSTF3* and AtGSTF12 to construct a protein interaction network to screen for suspicious genes (Figure 6; Table 1). The Y2H assay proved that *PsGSTF3* interacted with PsDFR (Figure 7). Qi demonstrated that PsMYB1 could transcriptionally activate the expression of PsDFR by directly binding to their promoters [61]. Coincidentally, MYBs bound to the promoter were confirmed to activate the transcription of GSTs in many species [27,30]. Therefore, we suspect that an MYB transcription factor can simultaneously regulate anthocyanin synthesis and transport genes. However, determining whether such a regulatory relationship exists in tree peony requires more in-depth research, which provides a direction for our next study.

Flower color has always been an important trait in ornamental horticultural plants, and tree peony is no exception. The synthetic pathway of anthocyanin has been well studied, but anthocyanin transport is also an indispensable step in the process of anthocyanin’s delivery into the vacuole for storage. Therefore, an in-depth understanding of anthocyanin transport is important for elucidating the mechanism underlying tree peony petal coloration. In this study, we used a variety of biological experimental methods to demonstrate that *PsGSTF3* encodes an important GST transporter of anthocyanin in tree peony petals. Additionally, *PsGSTF3* interacted with PsDFR, thereby enabling both to play a role together in tree peony petal coloration. Unfortunately, how *PsGSTF3* is regulated remains unclear in tree peony and also provides us with new ideas for future research. In short, anthocyanin accumulation is a complicated process affected by many factors, including the different genetic backgrounds of cultivars. Our study provides a basis for further investigations on the molecular mechanisms of anthocyanin transport in tree peony.

## 4. Materials and Methods

### 4.1. Plant Materials and Growth Conditions

The peony cultivar ‘Zhao fen’ was grown at the tree peony experimental site of Yuquan mountain in Beijing, China. The petals were collected in spring. The petals were quickly frozen in liquid nitrogen and stored at −80 °C.

Before sowing in the Murashige and Skoog (MS) solid medium, the *Arabidopsis tt19* mutant and tobacco seeds were disinfected by soaking in 70% ethanol for 2 min, soaking with 7% NaClO for 10 min, and then rinsing with aseptic water five times. Plant seedlings were grown in a controlled-environment room with the following conditions: 16 h light/8 h night photoperiod at 23 °C (*Arabidopsis*) and 25 °C (*tobacco*), with 60% relative humidity.

### 4.2. DNA and RNA Extraction, and Reverse Transcription

We conducted genomic DNA and total RNA extraction using a Plant Genomic DNA Kit (Tiangen, Beijing, China) and EASY Spin Plant RNA Extraction Kit (Aidlab, Beijing, China), respectively. The integrity and concentration of the genomic DNA and total RNA were verified using 1.0% agarose gel electrophoresis and a 2100 Bioanalyzer (Agilent Technologies, Palo Alto, CA, USA). Additionally, first-strand cDNA synthesis was performed using a cDNA reverse transcription kit (Transgen, Beijing, China), and the total RNA (1 μg) was studied using oligo (dT) primers according to the manufacturer′s instructions.

### 4.3. Gene Cloning and Sequence Analysis

The cDNA and DNA of *P. suffruticosa* ‘Zhao fen’ petals were used as the PCR templates. Then, the PCR product was cloned into the pMD19-T (Takara, Beijing, China) and confirmed by sequencing. The specific primer information is listed in Table S6. To explore the evolutionary relationships, a total of eight anthocyanin-related GST amino acid sequences (Table S3) were aligned through ClustalW, and a phylogenetic tree was constructed using the neighbor-joining (NJ) method in MEGA-X (version X, Mega Limited, Auckland, New Zealand), with 1000 bootstrap replications used to assess tree topology and reliability. Sequence homology and alignment were carried out with DNAMAN software (Version 7; Lynnon Biosoft, San Ramon, CA, USA).

### 4.4. Subcellular Localization Analyses

The coding sequence (CDS) of *PsGSTF3* without stop codons was cloned into PHG vectors (modified from PHB vector) to construct the 35S::PsGSTF3-GFP recombinant vector using a seamless cloning kit (Novoprotein, Suzhou, China). Then, the fusion constructs and negative control 35S::GFP vector were transformed into the *Agrobacterium strain* GV3101. Additionally, the ER-mCherry [62] was used to locate the fluorescent proteins in the ER, and then the 35S::PsGSTF3-GFP or 35S::GFP vector (1:1, *v/v*) was infiltrated into *N. benthamiana* leaves. The fluorescence signal was observed by laser confocal microscopy (Zeiss LSM 880 Meta, Jena, Germany). The excitation and emission wavelengths were 405 nm and 410–484 nm for blue fluorescence (DAPI), 488 nm and 500–540 nm for green fluorescence (EGFP), and 594 nm and 599–651 nm for red fluorescence (mCherry), respectively. The primers and restriction sites are listed in Table S7.

### 4.5. Overexpression of PsGSTF3 in The Arabidopsis tt19 Mutant

The full CDS of *PsGSTF3* was connected to the PHB vector by seamless cloning, and the *A. strain* GV3101 line containing 35S: PsGSTF3-PHB was introduced into the *A. thaliana* mutant *tt19* using the floral dipmethod [57]. The primers and restriction sites are listed in Table S7. T1 generation transgenic seeds were grown and screened on the MS medium containing 30 mg/L hygromycin B and 6% (*w/v*) sucrose. Seeds of the T3 generation were collected for follow-up experiments to confirm the results. The untransformed WT (Col-0) and *tt19* were used as the control. 

### 4.6. Virus-Induced Gene Silencing of PsGSTF3 in Peony

A 272 bp fragment of *PsGSTF3* (251–522 bp) was amplified and cloned into the pTRV2-GFP vector (modified from pTRV2 vector) to construct the pTRV2-PsGSTF3-GFP recombinant vector. The primers are listed in Table S7. Then, the pTRV1, pTRV2-GFP, and pTRV2-PsGSTF3-GFP plasmids were transformed into the *A. strain* GV3101, which was cultured in the LB medium (100 mg/L kanamycin; 25 mg/L rifampicin; 200 μM AS; 10 mM MES) at 28 °C for 12–16 h until an OD_600_ = 1.8 was reached. After centrifugation, the thallus was resuspended until reaching OD_600_ = 1.0 in an infiltration buffer containing 10 mM MES, 100 μM AS, and 10 mM MgCl_2_. Finally, pTRV1 was mixed with pTRV2-GFP and pTRV2-PsGSTF3-GFP in equal volumes respectively, and the mixed bacterial solution was allowed to stand for 4 h at room temperature in the dark [63,64,65,66,67,68]. To improve the infection efficiency, 0.01% Silwet L-77 was added to the infection liquid.

The perennial *P. suffruticosa* ‘Zhao fen’ that grows naturally in the field was used as the experimental material. The experimental treatment time was in mid-April. At this time, most buds of ‘Zhaofen’ were in the S1 (unpigmented tight bud) to S2 (slightly pigmented bud just before anthesis) periods of development. The injection experiment was carried out in the afternoon (15:00–17:00) with good weather conditions. The buds that met all the requirements were selected, and about 3 mL of infection solution was injected into the buds (internal and external) and pedicels. The infiltrated flower buds were given shaded treatment for 24 h and then allowed to grow normally in a natural state. After 7–10 days, photographs were taken, petals were collected for subsequent experiments, and the fluorescence signals of petals were imaged by a fluorescence microscope (Olympus BX51, Tokyo, Japan).

### 4.7. Tobacco Stable Transformation

The *A. strain* GV3101 line containing 35S: PsGSTF3-PHB was introduced into the tobacco using the leaf disc transformation method [69]. Firstly, the cultivation and infection treatment of sterile tobacco seedlings were performed using a previously described protocol [69], and the wild-type tobacco was used as a negative control. After infection, the leaves were consecutively carried out differentiation culture and rooting culture on the MS medium supplemented with relevant hormones (different concentrations of IBA and NAA) and antibiotics (hygromycin). Finally, the well-growing plantlets were transplanted into small flowerpots containing the substrate to maintain the original growth environment.

### 4.8. Gene Expression Analysis

The qRT-PCR experiment was performed using a SYBR Premix Ex TaqTM II Kit (TaKaRa, Beijing, China) on a LightCycler 480 system (Roche Applied Science, Penzberg, Germany). The experimental procedures were conducted in accordance with the manufacturer’s instructions and previous reports [61]. The PP2A gene was used as a reference gene for tree peony [70], and *Actin* was used for *Arabidopsis* and tobacco. The 2^−ΔΔCT^ method [51] was used for analysis and visualization of the qRT-PCR data generated by multiple technical replicates. The specific primer information is listed in Table S8.

### 4.9. Ultra-Performance Liquid Chromatography (UPLC) Analysis of Anthocyanin

Anthocyanin contents were detected by MetWare (http://www.metware.cn/, accessed on 23 December 2021) based on the AB Sciex QTRAP 6500 LC-MS/MS platform. The specific steps were as follows:

Sample preparation and extraction: Approximately 50 mg of tissue was transformed into a powder using liquid nitrogen, which was weighed and extracted with 0.5 mL methanol/water/hydrochloric acid (500:500:1, *v/v/v*). Then, the extract was vortexed for 5 min, exposed to ultrasound for 5 min, and centrifuged at 12,000 *g* under 4 °C for 3 min. Next, the residue was re-extracted by repeating the above steps again under the same conditions. The supernatants were collected, and filtrated through a membrane filter (0.22 μm, Anpel) before LC-MS/MS analysis.

UPLC Conditions: The sample extracts were analyzed using an UPLC-ESI-MS/MS system (UPLC, ExionLC™ AD; MS, Applied Biosystems 6500 Triple Quadrupole). The analytical conditions were as follows: UPLC—column, WatersACQUITY BEH C18 (1.7 µm, 2.1 mm × 100 mm); solvent system, water (0.1% formic acid)—methanol (0.1% formic acid); gradient program, 95:5 *v/v* at 0 min, 50:50 *v/v* at 6 min, 5:95 *v/v* at 12 min, hold for 2 min, 95:5 *v/v* at 14 min; hold for 2 min; flow rate, 0.35 mL/min; temperature, 40 °C; injection volume, 2 μL.

Qualitative and quantitative analysis: The MWDB (metware database) database was built based on standards and used to perform qualitative analysis on the mass spectrometry data. Quantification was accomplished using multiple reaction monitoring (MRM) analysis under triple quadrupole mass spectrometry. After obtaining the mass spectrometry analysis data from different samples, the chromatographic peaks of all targets were integrated, and quantitative analysis was performed using the standard curve.

### 4.10. Protein Interaction Network Prediction

According to the homologous relationship between *PsGSTF3* and AtGSTF12, we used the STRING 11.0 version software (https://string-db.org/, accessed on 23 December 2021) [71] to predict the proteins that may interact with *PsGSTF3*. The minimum required interaction score was set to medium confidence (0.400), and the maximum number of interactors was set to none/query proteins only.

### 4.11. Yeast Two-Hybrid Assay

The matchmaker GAL4 two-hybrid system was used for the Y2H assays. The CDS sequences of *PsGSTF3* and seven suspicious proteins (PsMATE, PsCHS, PsDFR, PsANS, PsUF3GT, PsUGT, and Ps3AT) were screened out via the protein interaction network and ligated to the pGBKT7 and pGADT7 vectors via homologous recombination technology. The primers are listed in Table S6. These recombinant vectors were confirmed by sequencing and co-transformed into AH109 yeast cells. Finally, the SD/-Trp-Leu medium was used to select transformed positive clones, and then the SD/-Trp-Leu-His-Ade + X-*α*-gal medium was used to select positive interacting clones.

### 4.12. Statistical Analysis

We performed statistical analyses with SPSS Statistics for Windows (Version 17.0; SPSS Inc., Chicago, IL, USA). All data represented the average and standard errors of three biological replicates. One-way analysis of variance (ANOVA) was used to evaluate the statistical significance of differences among means using the SPSS software. Single asterisks indicate significant differences at the levels of *p* < 0.05.

## 5. Conclusions

In this study, *PsGSTF3* from *P. suffruticosa* was successfully cloned and characterized. Our results showed that *PsGSTF3* contained GST-conserved domains and conformed to the typical gene structure characteristics of the Phi subfamily. At the same time, *PsGSTF3* was located on the nucleus and the ER membrane. *PsGSTF3* was found to have the ability to functionally complement the anthocyanin-deficient phenotype of the *Arabidopsis tt19* mutant but not the PA-deficient phenotype in the seed coat. The VIGS of *PsGSTF3* in tree peony petals can directly lead to a decrease in anthocyanin accumulation, while the heterologous overexpression of *PsGSTF3* can increase anthocyanin accumulation in tobacco petals. Additionally, the Y2H assay showed that *PsGSTF3* interacted with PsDFR, which together contribute to the coloration of petals. In summary, *PsGSTF3* played an irreplaceable role in the anthocyanin transport of tree peony.

## Figures and Tables

**Table 1:** The specific information and main functions of ten proteins in protein interaction networks.

Sr. No	*Arabidopsis thaliana*			*Paeonia suffruticosa*	
	Genes	GenBank Accession No.	Functions	Assembly Name	Genes
1	TT12	AT3G59030.1	Involved in the transportation of proanthocyanidin precursors into the vacuole	Unigene0083215	MATE
2	TT14	AT5G13930.1	Encodes CHS, a key enzyme involved in the biosynthesis of flavonoids	Unigene0034816	CHS
3	DFR	AT5G42800.1	Catalyzes the conversion of dihydroquercetin to leucocyanidin in ABP	Unigene0008084	DFR
4	LDOX	AT4G22880.1	Catalyzes the oxidation of leucoanthocyanidins into anthocyanidins	Unigene0024684	ANS
5	UF3GT	AT5G54060.1	Contributes to the last few anthocyanin biosynthetic steps	Unigene0078134	UF3GT
6	AT4G14090	AT4G14090.1	Encodes an anthocyanidin 5-*O*-glucosyltransferase	Unigene0008084	UGT
7	AT4G13780	AT4G13780.1	Its function is described as methionine-tRNA ligase activity	Unigene0007649	MetRS
8	AT5MAT	AT3G29590.1	Involved in the malonylation of the 5-*O*-glucose residue of anthocyanin	Unigene0095892	3AT
9	AT1G03495	AT1G03495.1	Involved in the acylation of the 6″ position of the 3-*O*-glucose residue of anthocyanin		
10	AHA10	AT1G17260.1	The plasma membrane H(+) ATPase of plants and fungi	Unigene0117747	AHA10

## Data Availability

The transcriptome data link is https://www.ncbi.nlm.nih.gov/sra/?term=SRP235658%20(SRP235658 (accessed on 23 December 2021).

## Supplementary Materials

The following are available online at https://www.mdpi.com/article/10.3390/ijms23031423/s1.
