# Supplementary material for: PsGSTF3, an Anthocyanin-Related Glutathione S-Transferase Gene, Is Essential for Petal Coloration in Tree Peony
Source: Int J Mol Sci. 2022 Jan 26;23(3):1423. doi: 10.3390/ijms23031423 (PMC8836093; doi:10.3390/ijms23031423)
Supplement: Supplementary file 1 [file ijms-23-01423-s001.zip › ijms-1546468-supplementary.pdf]

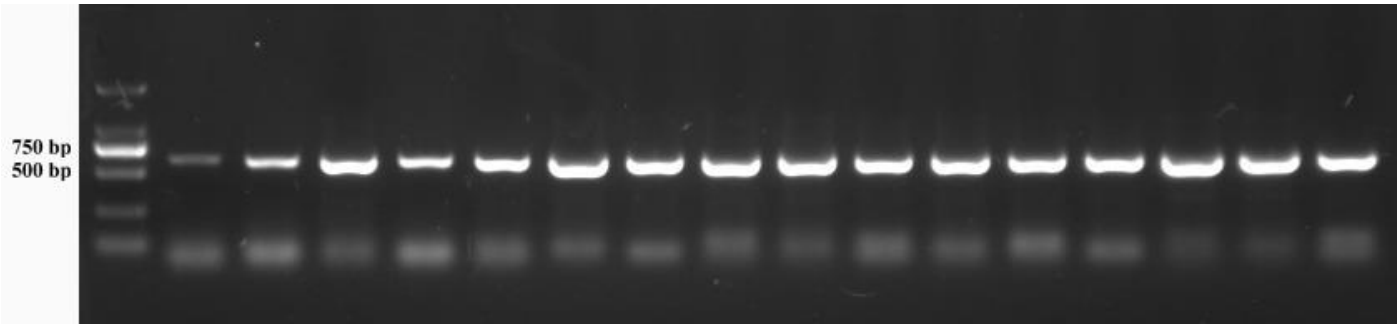

Figure S1. The electropherogram of positive PCR detection in 16 transgenic *Arabidopsis* lines.

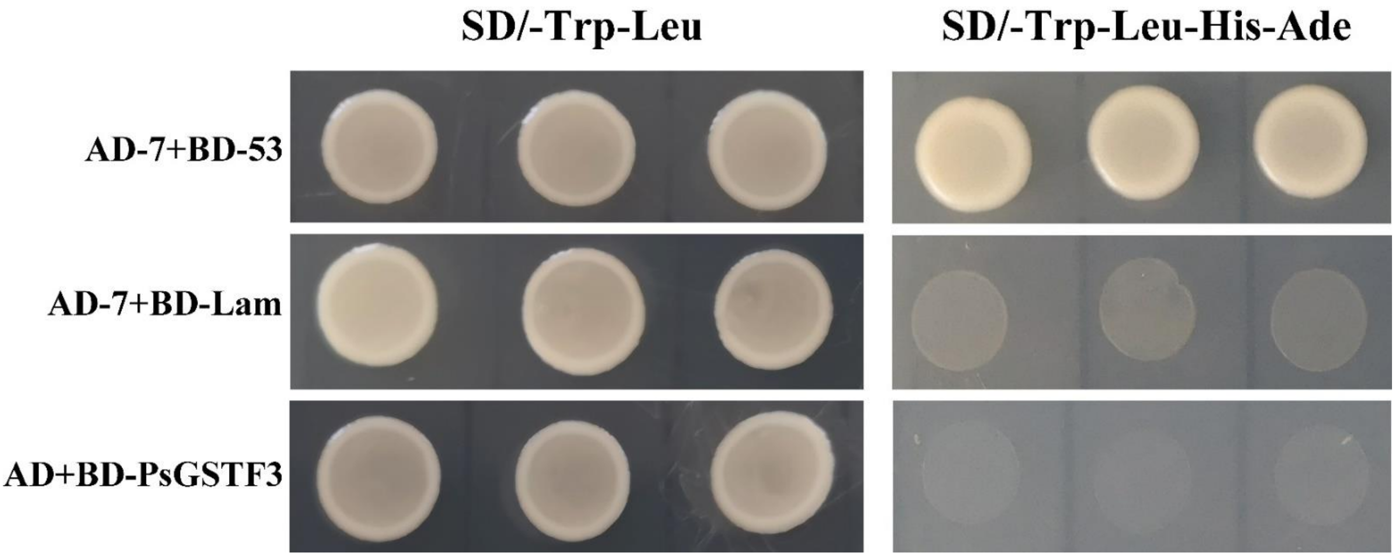

Figure S2. The autoactivation detection of PsGSTF3. The AD-T and BD-P53 was used as positive controls. The AD-T and BD-Lam was used as negative control.

Table S1. Summary of anthocyanin-related GSTs in different species

Species	Genes	Classes	Gene length(bp)	GenBank Accession No.	Aliases	Reference
Zea mays	*Bronze2*	Tau	2948 (DNA)	X81971.1	-	Marrs, 1995
Arabidopsis thaliana	*AtGSTF12*	Phi	1096 (mRNA)	NM_121728.4	AtTT19	Kitamura, 2004
Petunia hybrida	*PhAN9*	Phi	976 (mRNA)	Y07721.1	-	Alfenito, 1998
Glycine max	*GmGST26A*	Tau	987 (mRNA)	NM_001251510	HSP26-A	Li, 1997
Perilla frutescens	*PfGST1*	Phi	735 (mRNA)	AB362191.1	-	Yamazaki, 2008
Senecio cruentus	*ScGST3*	Phi	639 (mRNA)	-	-	Jin, 2013
Dianthus caryophyllu	*DcGSTF2*	Phi	5590 (DNA)	AB688111.1	-	Sasaki, 2012
Cyclamen persicum	*CkmGST3*	Phi	642 (mRNA)	AB682678.1	-	Kitamura, 2012
Ipomoea batatas	*IbGSTF4*	Phi	651 (mRNA)	MG873448	-	Kou, 2019
Litchi chinensiss	*LcGST4*	Phi	645 (mRNA)	KT946768.1	-	Hu, 2016
Malus domestica	*MdGSTF6*	Phi	1136 (mRNA)	AEN84869	-	Jiang, 2019
Prunus persica	*PpGST1*	Phi	-	-	-	Zhao, 2019
Vitis vinífera	*VviGST4*	Phi	642 (mRNA)	NM_001280940.1	-	Pérez-Díaz, 2016
Actinidia chinensis	*AcGST1*	Phi	601 (mRNA)	MK238757.1	-	Liu, 2019
	CsGSTa	Phi	642 (mRNA)	MK431867.1	-	
Camellia sinensis	*CsGSTb*	Tau	660 (mRNA)	MK431868.1	-	Liu, 2019
	CsGSTc	Tau	711 (mRNA)	MK431869.1	-	
Fragaria vesca	*FvRAP*	Phi	759 (mRNA)	-	-	Luo, 2018
***Lilium* spp.**	*LhGST*	Phi	684 (mRNA)	MK426728	-	Cao, 2020
Gossypium hirsutum	*GhGSTF12*	Phi	-	GH_D07G0816	-	Shao, 2021
Raphanus sativus	*RsGST1*	Phi	-	RSG01330.t1	-	Lai, 2021
Euphorbia pulcherrima	*Bract1*	Phi	684 (mRNA)	-	-	Vilperte, 2021

Table S2. Sequences of PsGSTF3

>PsGSTF3 genomic DNA sequence
ATGGTAGTTAAGGTGTATGGCTCAGCTAAAGCGGCTTGTCCACAAAGAGTGATGGTTTGCCTTCTGGAAAAGGAGGTGGA
ATTTGAAATTATACATGTTGATCTTGAATCTGGAGAGCACAAAAAGCCAGATTTCCTTGCTCGGCAGGTAGAATTCAGGCTT
GTACCCCCATGAAAAATATGGCTTTCAAGTTTCTAACATGTAGTTGTTCGTTTTTCTTGTAGCCGTTTGGGCAAGTTCCAGCC
ATCGAGGATGGTGACTTGAAACTTTTTGGTAAGAGCATTGATTATATATAATGATATTTTGTGGATTCATTAAGATAAATAAT
GGATTTCTGGTTTTGGTGCTTTCTAAAAGCAGAGTCTGTGTTATTGAGTTCACTAACATGTATCTATACAAAATGTAAAAAAA
TTTCCACAGAATCCAGGGCAATCATAAGATACTATGCAGCCAAGTTTGATAACCGCGGTTCAAACCTGTTGGGAACCACTT
TGGAAGAGAGAGCTTTGATGGATCAATGGCTAGAAGTTGAAGCCCACAATTTCAATGATTTGGTTTACACTATTGTGCTTCA
GATCGTAATTCTCCCCCGTATGGGACAAGGTACTGACTTGGCATTAGTCCGCACCTGTGAAGAAAAGCTTGAGAAAGTGCT
TGATGTGTACGAGCAAAAGTTGTCAAAGAGCAGCTACCTGGCCGGAGACGATTTCACACTCGCTGATCTCAGTCATCTTCC
TGGTCTCAGATACCTCATAAATGAAGCTGGAAAAGGATACCTGGTGACCGTGAGGAAGAATGTGAATGCATGGTGGGAGA
ATATATCAAATCGTCCTTCTTGGAAGAAATTAATGAATCTTGTTAATTAA
Note: The sequence in the shade represents introns.

>PsGSTF3 cDNA sequence
ATGGTAGTTAAGGTGTATGGCTCAGCTAAAGCGGCTTGTCCACAAAGAGTGATGGTTTGCCTTCTGGAAAAGGAGGTGGA
ATTTGAAATTATACATGTTGATCTTGAATCTGGAGAGCACAAAAAGCCAGATTTCCTTGCTCGGCAGCCGTTTGGGCAAGTT
CCAGCCATCGAGGATGGTGACTTGAAACTTTTTGAATCCAGGGCAATCATAAGATACTATGCAGCCAAGTTTGATAACCGC
GGTTCAAACCTGTTGGGAACCACTTTGGAAGAGAGAGCTTTGATGGATCAATGGCTAGAAGTTGAAGCCCACAATTTCAAT
GATTTGGTTTACACTATTGTGCTTCAGATCGTAATTCTCCCCCGTATGGGACAAGGTACTGACTTGGCATTAGTCCGCACCT
GTGAAGAAAAGCTTGAGAAAGTGCTTGATGTGTACGAGCAAAGGTTGTCAAAGAGCAGCTACCTGGCCGGAGACGATTTC
ACACTCGCTGATCTCAGTCATCTTCCTGGTCTCAGATACCTCATAAATGAAGCTGGAAAAGGATACCTGGTGACCGTGAGG
AAGAATGTGAATGCATGGTGGGAGAATATATCAAATCGTCCTTCTTGGAAGAAATTAATGAATCTTGTTAATTAA

>PsGSTF3 amino acid sequences
MVVKVYGSAKAACPQRVMVCLLEKEVEFEIIHVDLESGEHKKPDFLARQPFGQVPAIEDGDLKLFESRAIIRYYAAKFDNRGSNLLG
TTLEERALMDQWLEVEAHNFNDLVYTIVLQIVILPRMGQGTDLALVRTCEEKLEKVLDVYEQRLSKSSYLAGDDFTLADLSHLPGLR
YLINEAGKGYLVTVRKNVNAWWENISNRPSWKKLMNLVN

Table S3. The anthocyanin-related GST amino acid sequences have been confirmed in other species

>MdGSTF6
MVVKVYGPVMAACPQRVMVCLLEKGVNFEIVDVDLEAGEHKRPEFLTRQPFGQVPVVEDGDFRLFESRAIIRYYAAKYAGRGPNL
LGTTLEEKAVVDQWLEVEGHNFNDLVYTLVLQLLVLPRMGQRGDAALINACEEKLEKVFDVYEERLSKSKYLAGETFTLADLSHLP
GIRYLIDEAKLGHLVTGRKKVNAWWEDISNRPSWKKLMQLASDY
>FvRAP
MQFSSHEDEDEDEGNRLLNDFLCSDTTYYRPVFLYSSQMVLKVYGPVRAACPQRVMVCLLELGVEFEIVPVDLQAGEQKQPHILA
RQPFGQVPAIEDGDFKLFESRAIVRYYAAKYAERGPNLLGTTLEEKALVDQWLEVESHNFNDLVFTVVLQLVILPSMGQPGDLALV
RSCEEKLKKVFDVYEERLSKSTYLAGNYFSLADLSHLPAIRFLVDEFKMGHLITERKNVNAWWKDISNRPAWKKLMKLAQY
>VviGST4
MVMKVYGPVRAACPQRVLACLVEKGVEFEVVHVDLDSGEQKRPDFLLRQPFGQVPVVEDGDFRLFESRAIVRYIAAKYAEQGPD
LLGKSLEEKAVVDQWLEVEAHNFNELVYTLVMQLVILPRMGERGDLALAHTCEQKLEKVFDVYEQRLSKSRYLAGDSFTLADLSHL
PAIRYLVKEAGMAHLVTERKSVSAWWEDISNRAAWKKVMELAA
>CkmGST3
MVVKVYGPATAGCPQRVIACLFELDVDFEIIHVDLESGEHKKPDFLLRQPFGQVPAIEDGDFRLFESRAIMRYYAAKYSEKNPDLQG
STLEEKALVDQWLEVESHNFNDLVYTLVLHLMVFPQMGKRSDMQLVQECESKLEKVFDIYEERLSKSNYLAGKLFTLADLSHLPSIT
FLMGEGGLGHMVRNRKNVNSWWMDISSRPSWKKVRKLMD
>PhAN9
MVVKVHGSAMAACPQRVMVCLIELGVDFELIHVDLDSLEQKKPEFLVLQPFGQVPVIEDGDFRLFESRAIIRYYAAKYEVKGSKLTG
TTLEEKALVDQWLEVESNNYNDLVYNMVLQLLVFPKMGQTSDLTLVTKCANKLENVFDIYEQRLSKSKYLAGEFFSLADLSHLPSL
RFLMNEGGFSHLVTKRKCLHEWYLDISSRDSWKKVLDLMMKKISEIEAVSIPAKEEAKV
>DcGSTF2
MGVIKVYGAKKAACPQRVIACLIELGIDYDVIFVDLESGHHKQPDFLALQPFGQVPVIEDGDFRLYESRAIIRYLATKYSPKNSLNNN
LLGTNVEEKAFVDQWLEVEAHHFNQQVYNIVLELIVFPKMGKQTDISYVHSLEKKLANVLDVYEERLSKSKYLAGDFFTLADLSHLP
GLRYLTTEAKMPHLVEDLKCVNAWWTDISSRPAWKKVLSLDN
>AtGSTF12
MVVKLYGQVTAACPQRVLLCFLEKGIEFEIIHIDLDTFEQKKPEHLLRQPFGQVPAIEDGDFKLFESRAIARYYATKFADQGTNLLGK
SLEHRAIVDQWADVETYYFNVLAQPLVINLIIKPRLGEKCDVVLVEDLKVKLGVVLDIYNNRLSSNRFLAGEEFTMADLTHMPAMG
YLMSITDINQMVKARGSFNRWWEEISDRPSWKKLMVLAGH

Table S4. Determination of anthocyanin content of tree peony between pTRV1/pTRV2-GFP and pTRV1/pTRV2-PsGSTF3-GFP treated groups via UPLC

**Compounds**	**Class**	**CK-1**	**CK-2**	**CK-3**	**VIGS-1**	**VIGS-2**	**VIGS-3**
Cyanidin-3,5,3'-O-triglucoside	Cyanidin	0.116773638	0.097501934	0.097660182	0.091760746	0.086762049	0.068153955
Cyanidin-3,5-O-diglucoside	Cyanidin	0.217203526	0.157175524	0.145562918	0.149296229	0.122106675	0.125556795
Cyanidin-3-O-sophoroside	Cyanidin	0.8484375	0.699161067	0.697700101	0.439306569	0.518646905	0.444588235
Cyanidin-3-O-(6-O-p-coumaroyl)-glucoside	Cyanidin	0.182440104	0.118745266	0.097388045	0.094874493	0.099179875	0.088471197
Cyanidin-3-O-galactoside	Cyanidin	3.79887821	3.50199552	3.69140831	2.17141119	2.23924178	1.94690669
Cyanidin-3-O-rutinoside-5-O-glucoside	Cyanidin	0.002803365	0.002959275	0.003392847	0.000591488	0.000275923	0.001738815
Pelargonidin-3,5-O-diglucoside	Pelargonidin	195.139623	187.028711	199.404458	76.8136659	70.8888889	84.5233266
Pelargonidin-3-O-(6-O-p-coumaroyl)-glucoside	Pelargonidin	169.984776	159.087966	148.953394	139.847121	154.61706	150.920892
Pelargonidin-3-O-glucoside	Pelargonidin	4.28317308	5.9380167	5.41339412	0.350214923	0.309046179	0.649567951
Pelargonidin-3-O-rutinoside	Pelargonidin	0.047996595	0.028742008	0.031952381	0.024090835	0.033475499	0.01953355
Pelargonidin-3-O-sophoroside	Pelargonidin	1.08213942	0.995115048	1.03764134	0.761248986	0.761351079	0.694399594
Pelargonidin-3-O-5-O-(6-O-coumaroyl)-diglucoside	Pelargonidin	0.223229167	0.102395642	0.064240122	0.053168897	0.097231902	0.064039351
Peonidin-3-O-(6-O-p-coumaroyl)-glucoside	Peonidin	23.5651042	6.76489513	3.88348531	9.45995539	13.2415205	12.1854767
Peonidin-3-O-glucoside	Peonidin	0.082252404	0.075719405	0.057574266	0.02121249	0.020908046	0.027973022
Petunidin-3-O-glucoside	Petunidin	0.897740385	0.567774384	0.500285714	0.992230333	0.90374874	1.05972211
Delphinidin-3-O-(6-O-malonyl-beta-D-glucoside)	Delphinidin	0.011621555	0.011345775	0.010132422	0.018051237	0.01338534	0.015051724
Delphinidin-3-O-galactoside	Delphinidin	0.017654868	0.009945551	0.014525775	0.003704542	0.006305283	0.006067546
Delphinidin-3-O-glucoside	Delphinidin	0.062876002	0.034369375	0.02950922	0.068275345	0.043402299	0.070381947
Malvidin-3,5-O-diglucoside	Malvidin	0.008886899	0.008653614	0.009422533	0.006950345	0.007437124	0.00737428

Note: CK: the petals injected with pTRV1/pTRV2-GFP; VIGS: the petals injected with pTRV1/pTRV2-PsGSTF3-GFP.

Table S5. Determination of anthocyanin content of tobacco between wild type and transgenic lines via UPLC

Compounds	Class	WT-1	WT-2	WT-3	OE-1	OE-2	OE-3
Cyanidin-3-O-glucoside	Cyanidin	2.7043032	13.4946237	4.88285095	61.6345371	48.1622307	53.3467539
Cyanidin-3-O-arabinoside	Cyanidin	N/A	N/A	N/A	0.0496877545	0.0499366286	0.0445395068
Cyanidin-3-O-xyloside	Cyanidin	0.00473746546	0.0284946237	0.00913565663	0.184632093	0.178200253	0.173628586
Cyanidin-3-O-sambubioside	Cyanidin	0.0538886696	0.211290323	0.0765898799	0.985609557	1.00633714	0.968797182
Cyanidin-3-O-rutinoside	Cyanidin	29.2143703	84.4086022	39.7716086	232.690741	233.460076	223.955712
Cyanidin-3-O-(6-O-malonyl-beta-D-glucoside)	Cyanidin	0.134622977	0.181989247	0.172081118	1.06434971	1.19391635	1.11977856
Cyanidin-3-O-sophoroside	Cyanidin	0.0244769049	0.0491935484	0.0318960425	0.20961173	0.231178707	0.218419728
Cyanidin-3-O-rutinoside-5-O-glucoside	Cyanidin	0.66127122	2.08064516	0.990352432	8.82432799	13.0038023	11.172622
Delphinidin	Delphinidin	N/A	N/A	N/A	0.0325821341	0.0306717364	0.033467539
Delphinidin-3-O-sophoroside	Delphinidin	0.099684169	0.115322581	0.0968694625	0.15557969	0.202534854	0.134121792
Delphinidin-3-O-rutinoside-5-O-glucoside	Delphinidin	95.5388867	115.053763	103.169915	153.950584	166.539924	136.889784
Delphinidin-3-O-rutinoside	Delphinidin	10.9553889	12.9301075	11.7542823	17.893022	18.9353612	17.4635128
Delphinidin-3-O-galactoside	Delphinidin	0.116462692	0.149193548	0.130143729	0.269074124	0.30418251	0.301962758
Delphinidin-3-O-arabinoside	Delphinidin	0.0491512041	0.0766129032	0.0376058279	0.0589193592	0.0798479087	0.0530951183
Delphinidin-3-O-(6-O-malonyl)-glucoside-3'-glucoside	Delphinidin	0.186932491	0.271505376	0.171293562	0.404561499	0.474017744	0.480624056
Delphinidin-3-O-(6-O-malonyl-beta-D-glucoside)	Delphinidin	2.09238058	2.45430108	2.06733609	4.04561499	4.84157161	5.00754907
Pelargonidin-3-O-rutinoside	Pelargonidin	55.6652191	179.83871	75.0147667	795.547108	839.036755	757.423251
Pelargonidin-3-O-glucoside	Pelargonidin	0.256612712	1.05107527	0.360307147	5.67472169	5.22179975	4.68042275
Pelargonidin-3-O-galactoside	Pelargonidin	0.256612712	0.39516129	0.28548927	0.374694542	0.43599493	0.357322597
Pelargonidin-3-O-arabinoside	Pelargonidin	0.0519147256	0.0470430108	0.0470565072	0.112136845	0.180481622	0.137141419
Pelargonidin-3-O-(6-O-malonyl-beta-D-glucoside)	Pelargonidin	0.144097908	0.663978495	0.2087025	4.61580234	4.7148289	4.20231505
Pelargonidin	Pelargonidin	N/A	N/A	N/A	0.169427097	0.178453739	0.150226472
Pelargonidin-3,5-O-diglucoside	Pelargonidin	N/A	N/A	N/A	0.066793375	0.118631179	0.090840463
Petunidin-3-O-(6-O-malonyl-beta-D-glucoside)	Petunidin	2.68456376	2.68817204	2.63831463	3.58403475	4.43599493	4.65525918
Petunidin-3-O-galactoside	Petunidin	0.183379392	0.0470430108	0.131521953	0.14580505	0.192141952	0.109964771
Petunidin-3-O-rutinoside	Petunidin	0.523095144	0.61827957	0.488285095	0.597339126	0.709759189	0.699547056
Petunidin-3-O-sambubioside-5-O-glucoside	Petunidin	N/A	0.0279569892	0.0192754479	0.0328536519	0.0261089987	0.0440362355
Petunidin-3-O-glucoside	Petunidin	0.0238847217	0.00298387097	0.0271707029	0.00204724409	0.0160963245	0.0161550075
Peonidin-3-O-rutinoside	Peonidin	2.28977497	7.66129032	3.26835991	37.740972	48.9226869	40.2617011
Peonidin-3-O-(6-O-malonyl-beta-D-glucoside)	Peonidin	0.0396762732	0.0704301075	0.0374089388	0.227260386	0.191381496	0.224710619
Peonidin-3-O-glucoside	Peonidin	N/A	N/A	N/A	0.0464295411	0.0400506971	0.0415198792
Peonidin	Peonidin	0.0949467035	0.0825268817	0.10218547	0.235948955	0.306717364	0.235279316
Malvidin-3-O-(6-O-p-coumaroyl)-glucoside	Malvidin	0.104026846	0.0994623656	0.112817484	0.127341841	0.163498099	0.157523905
Malvidin-3-O-arabinoside	Malvidin	0.0546782471	0.086827957	0.0470565072	0.120282378	0.11026616	0.0903371917

Note: WT: the petals of wild type; OE: the petals of transgenic lines.

Table S6. Primer information used for full-length amplification of *PsGSTF3*

Primer name	Nucleotide sequence	Purpose
Q-PsGSTF3-F	ATGGTAGTTAAGGTGTATGG	Full-length amplification
Q-PsGSTF3-R	TTAATTAACAAGATTCATTAATTTC	
M13-47	CGCCAGGGTTTTCCCAGTCACGAC	Universal primer of pMD19-T vector
RV-M	GAGCGGATAACAATTTCACACAGG	

Table S7. Primer information used for vector construction

Primer name	Nucleotide sequence	Purpose
PsGSTF3-PHB-F	ACCAGTCTCTCTCTCAAGCTTATGGTAGTTAAGGTG	for heterologous overexpression
PsGSTF3-PHB-R	GATACGAACGAAAGCTCTAGATTAATTAACAAGATT	
PsGSTF3-PHG-F	TCTCTCTCAAGCTTGGATCCATGGTAGTTAAGGTGTATGGCTC	for Subcellular localization
PsGSTF3-PHG-R	CGGGTCATGAGCTCCTGCAGATTAACAAGATTCATTAATTTC	
PsGSTF3-pTRV2-F	GTGAGTAAGGTTACCGAATTCACCTGTTGGGAACCACTTTG	for VIGS
PsGSTF3-pTRV2-R	CGTGAGCTCGGTACCGGATCCTCTGAGACCAGGAAGATGACT	
BD-PsGSTF3-F	AGGCCGAATTCCCGGGGATCCATGGTAGTTAAGGTGTATGGC	for Y2H
BD-PsGSTF3-R	CTAGTTATGCGGCCGCTGCAGTTAATTAACAAGATTCATTAA	
AD-PsMATE-F	GTGGGCATCGATACGGGATCCATGGGTTCCGTACGGTACCA	
AD-PsMATE-R	ATTCATCTGCAGCTCGAGCTCTTATATGTTGTCCACAAACAAGGCC	
AD-PsANS-F	GCCATGGAGGCCAGTGAATTCATGGTGAATTCAGTAGCTCC	
AD-PsANS-R	ATTCATCTGCAGCTCGAGCTCTCAATTCTTAAACTCTTCTTGGG	
AD-PsUF3GT-F	GCCATGGAGGCCAGTGAATTCATGGCTACAAATAGCATAG	
AD-PsUF3GT-R	ATTCATCTGCAGCTCGAGCTCTTATTCCAGCATTTGTTTCATG	
AD-DFR-F	GTACCAGATTACGCTCATATGATGCGACTTCTGGAACATGG	
AD-DFR-R	ATTCATCTGCAGCTCGAGCTCTTAGATTGTGTCATTAACATGG	
AD-CHS-F	GCCATGGAGGCCAGTGAATTCATGGCTTCAGTTGAAGAAATTAG	
AD-CHS-R	ATTCATCTGCAGCTCGAGCTCTCACTCACTGATTGTAATTGCAGG	
AD-PsUGT-F	GTACCAGATTACGCTCATATGATGGCGCACCACCACTTCCT	
AD-PsUGT-R	CAGCTCGAGCTCGATGGATCCTCACAAATCACAACCTTTCCC	
AD-Ps3AT-F	GTACCAGATTACGCTCATATGATGTCTCTCGTCCACACAGT	
AD-Ps3AT-R	CAGCTCGAGCTCGATGGATCCTCAACCTACAACTTTCAAACC	
GAL4-AD	TACCACTACAATGGATG	Universal primer of pGADT7
3'AD	AGATGGTGCACGATGCACAG	
GAL4-BD	TCATCGGAAGAGAGTAG	Universal primer of pGBKT7
3'BD	TAAGAGTCACTTTAAAATTTGTATAC	

The restriction sites are underlined.

Table S8. Primer information used for qRT-PCR

Gene name	Forward (5' to 3')	Reverse (5' to 3')
PsCHS	CAAAGGGTCACGAGTTCTA	CACGCAAGTGTCCATCA
PsCHI	CCCACCTGGTTCTTCTATT	CAGCACAGCCTCGGAAAGTTGTT
PsF3H	CAACGAAATCCCAATCAT	TTGTCATCAGGCGGTAGA
PsF3'H	TGTTCACGGCAGGGACT	TTTCTTGTTGGGCTTGG
PsDFR	ATCGGTTCATGGCTTGT	AATGGGTATCCGCTTTT
PsANS	GAAGATTGCTGGGTATGG	CTGAGTTGCTTGGCGTA
PsUFGT	CTGGAGGTATTGTAGGGC	GTTCAAGTTTCCGACGAG
PsGSTF3	TGATGGTTTGCCTTCTGG	TTATGATTGCCCTGGATT
PsPP2A	TGATTACTTGCCCCTCACAGCC	CATAACAGGTCGCACATTGGTC
AtActin	CTCCTGAAGAGCACCCTG	CCCTCGTAGATTGGCACA
